# Barriers and facilitators for consuming a plant-based diet in patients with knee osteoarthritis: a qualitative study

**DOI:** 10.3389/fnut.2026.1743219

**Published:** 2026-03-13

**Authors:** Sabine Chmelar, Ursula Trübswasser, Tatjana Aubram, Gabriele Leitner, Andrea Haas, Oliver Neubauer, Karl-Heinz Wagner, Barbara Wondrasch, Elisabeth Höld

**Affiliations:** 1 Institute of Health Sciences, University of Applied Sciences St. Pölten, St. Pölten, Austria; 2 Vienna Doctoral School of Pharmaceutical, Nutritional and Sport Science (PhaNuSpo), University of Vienna, Vienna, Austria; 3 Institute for Innovation Systems, University of Applied Sciences St. Pölten, St. Pölten, Austria; 4 Department for Health Sciences, Medicine and Research, University for Continuing Education Krems, Krems, Austria; 5 Research Platform Active Ageing, University of Vienna, Vienna, Austria; 6 Department of Nutritional Sciences, University of Vienna, Vienna, Austria

**Keywords:** focus group, nutritional therapy, osteoarthritis, plant-based diet, qualitative

## Abstract

**Introduction:**

A predominantly plant-based dietary concept was developed in the course of the NUMOQUA study (Nutrition and Movement to Improve Quality of Life in Patients with Knee Osteoarthritis) as part of the interdisciplinary therapeutic intervention for patients with knee osteoarthritis (OA). This study included focus group discussions (FGDs), which aimed to identify perceived barriers and facilitators for following these dietary recommendations.

**Methods:**

In total, 4 FGDs were held with a total of 24 participants aged 50–75 years with mild to moderate knee OA, who belonged to the intervention group of the NUMOQUA study and followed the plant-based diet. FGDs were recorded, transcribed, and coded using an adapted version of the socio-ecological framework in MAXQDA (Max Qualitative Data Analysis) software. Thematic areas were identified by collating data relevant to each code, code group, and the four spheres of influence (individual, interpersonal and social, environmental, and policy).

**Results:**

Barriers for adopting the dietary recommendations primarily involved the limited availability of suitable options when eating out, adherence to traditional eating habits, and prioritizing family members’ preferences. Facilitators included family support, goal-setting, establishing a routine, individual approaches, and motivation derived from improved physical health.

**Discussion:**

The results reflect the diversity of influencing factors at the individual, interpersonal and social, and environmental levels, and provide important information for future dietary interventions for those affected by knee OA and other non-communicable diseases, particularly as cardiovascular diseases and type 2 diabetes.

**Clinical trial registration:**

https://clinicaltrials.gov/study/NCT05955300, identifier NCT05955300.

## 1 Introduction

Osteoarthritis (OA) is the most prevalent arthritic disease worldwide, with an increasing prevalence with age (1). Overall, OA affects 7.6% of the global population, rising to 23.2% in the group aged 50–69 years, and 38.4% in people aged 70 years and older (2). It is characterized by pain, effusion, and stiffness, leading to functional decline and loss of quality of life (3). OA is the 11th highest global cause of disability, resulting in significant morbidity and costs to the healthcare system (3, 4). The direct costs of OA account for 1–2.5% of gross national product per year in countries with established market economies, such as in North America or Europe. Indirect costs, such as those resulting from lost workplace productivity and early retirement, are also substantial and vary from $1.2 billion (Spain) to $12.7 billion (US) per year (5). These costs will likely increase substantially, because cases of knee OA, which are the most common form of OA, are expected to increase globally by 74.9% from 2020 to 2050 (2). The main factors contributing to this increase are rising life expectancy, population growth, and the increasing prevalence of obesity, with the impact varying by region (2, 6). Furthermore, people under the age of 45 are also being diagnosed more frequently (7). Therefore, OA is the fastest-growing cause of disability worldwide (8).

Given the substantial and growing burden of OA, understanding modifiable biological pathways that drive OA has become increasingly important, with inflammation emerging as a central mechanism in the OA pathogenesis (9). Triggered by tissue damage and metabolic dysfunction, inflammatory processes within the joint, accompanied by the production of inflammatory mediators such as cytokines, growth factors, and adipokines (local inflammation), may trigger the development of chronic low-grade inflammation (systemic inflammation) (10, 11). Factors that contribute to chronic low-grade inflammation include excessive energy intake, micronutrient deficiency, obesity, and abnormal metabolites, such as excessive production of reactive species (12). Reducing overweight or obesity is the most evidence-based dietary intervention in OA, as this reduces the load on the joints and systemic inflammatory processes simultaneously (13). More recent data show that the inflammatory potential of dietary patterns also influences OA and its progression (14, 15). Diets with a higher inflammatory potential, such as the Western diet—characterized by a high intake of fat, sugar, salt, processed food, and a low intake of plant-based foods—can increase the incidence and the progression of symptomatic OA (16, 17). In contrast, anti-inflammatory diets, such as the Mediterranean diet—including high consumption of fish and plant-based foods like vegetables, fruits, whole grains, and legumes—can lead to improved physical function and reduced pain, while also positively influencing OA progression and inflammatory markers (18–20). In a recent meta-analysis, Limongi et al. confirmed an association between adherence to the Mediterranean diet and a slight reduction in pain risk for patients with knee OA (21). Therefore, anti-inflammatory lifestyle behavior change guided by dietitians and exercise therapy should be part of first-line interventions in the management of OA and hold the promise of increasing a patient’s inflammatory threshold, reducing the rate of diseases progression, reducing weight, and maximizing health by minimizing a patient’s risk or manifestation of other lifestyle-related comorbidities (22, 23) without side-effects.

A plant-based therapeutic diet should focus on foods rich in polyphenols and vitamins like fruit or vegetables, high-quality fatty acids of vegetable oils and fish, and a high intake of fiber (24–26). Adopting a plant-based diet (PBD) or therapeutic diet is accompanied by various barriers, such as concerns about meeting nutritional requirements with a PBD, personal dietary habits related to animal-source foods, and a lack of knowledge regarding adequate food selection in a PBD (27). Additionally, limitations in time and money, dietary habits, and taste preferences are mainly responsible for the (non-)adherence to a therapeutic diet (28–30). Recommendations to adopt an anti-inflammatory therapeutic diet, like a plant-based Mediterranean diet, for OA (20, 31) might be less attractive for patients as it does not correspond with regional preferences, culture, traditions, or the limited availability of recommended Mediterranean food (32). In order to tackle these limitations, the New Nordic Diet was developed in the early 2000s by experts to address health and sustainability concerns, food culture, and gastronomic potential in Scandinavia. The basic principle of the New Nordic Diet is to increase calories from plant-based food sources while reducing those from animal products. Another important recommendation is to include more food from the sea, lakes, and the wild countryside. Most importantly, these principles and guidelines can be applied to any region (33, 34), including Austria.

The NUMOQUA study (Nutrition and movement to improve quality of life in patients with knee osteoarthritis) developed an anti-inflammatory, plant-based, therapeutic diet for patients with OA under the working title “Austrian OA Cuisine,” which was implemented within a randomized controlled trial (RCT) (35). According to a broader definition of a PBD (36), the dietary concept emphasized a primarily PBD with more than 70% of foods derived from plants and limited amounts of meat, dairy, eggs, and fish. The key components of the diet were whole grains, legumes, vegetables, fruit, nuts, fish, and plant oils that were rich in omega-3 fatty acids. The diet was based on the average daily intake of the dietary components in the New Nordic Diet (37) combined with the food-based dietary guidelines of Austria, valid at that time (38), the guidelines for knee OA (39, 40), typical Austrian recipes (41, 42), and the principles of a sustainable diet (43). The NUMOQUA study provides valuable findings regarding the combination of this anti-inflammatory PBD with an evidence-based training program for patients with knee OA. Quantitative data evaluates its impact on quality of life, nutritional status, inflammatory status, and oxidative stress parameters.

This study is focused on the qualitative part of the NUMOQUA study and aims to identify perceived barriers and facilitators to following the “Austrian OA Cuisine” in participants’ daily lives. The results of the qualitative analysis will improve the understanding of the acceptance and implementation of the “Austrian OA Cuisine.” As anti-inflammatory therapeutic diets based on plant-based foods are essential for managing other non-communicable diseases, such as cardiovascular diseases or type 2 diabetes, the outcomes of this study can be transferred to interventions for these diseases. A better understanding of the barriers and facilitators to follow a PBD can help to promote a PBD in the general population and meet several sustainable development goals (SDGs). These include supporting good health and wellbeing (SDG3), responsible consumption and production (SDG 12), and climate action (SDG13) (44).

## 2 Methods

### 2.1 Study design

**Figure 1:**
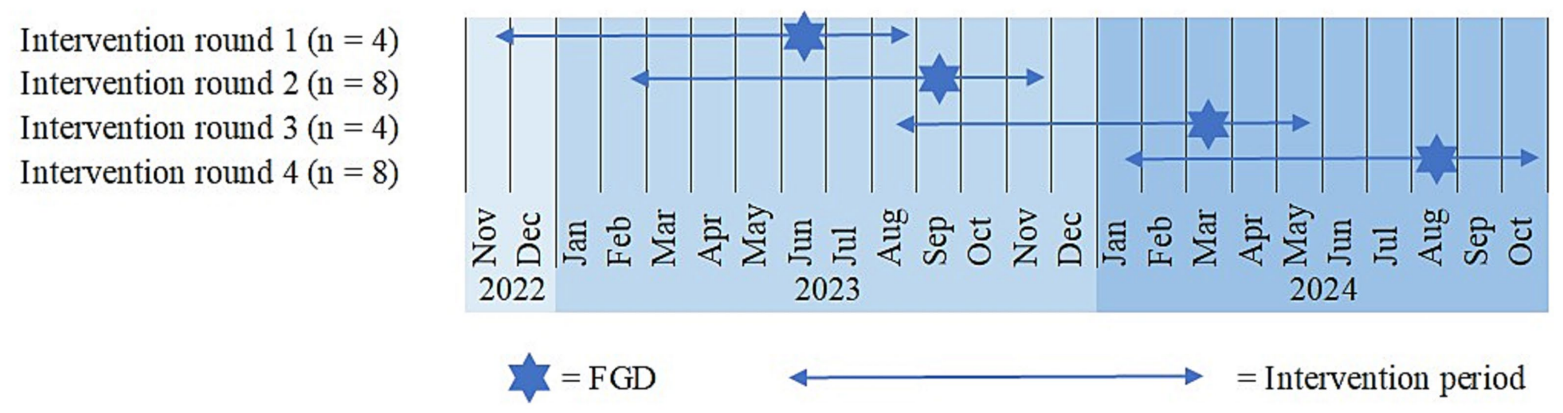
Timeline of intervention rounds and FGDs (*n* = participants in the FGDs).

The NUMOQUA study examined the implementation of the “Austrian OA Cuisine” combined with the evidence-based training program GLA:D^®^ (Good Life with Osteoarthritis in Denmark^®^) (45) in Austrian patients with mild to moderate knee OA and the effects on quality of life, nutritional and inflammatory status, and oxidative stress parameters. A detailed study protocol of the randomized controlled trial has been published previously (35). In brief, participants in the intervention group received dietetic group training in the first 6 weeks and comprehensive individual counseling by a dietitian until 9 months after the start of the study. The control group received general information about a healthy lifestyle. Both groups participated in the GLA:D^®^ training program during the first 6 weeks of the study. Measurements at baseline and at 4 follow-up dates included nutritional, inflammatory, and oxidative stress parameters. Furthermore, anthropometric and behavioral parameters and clinical data were assessed. An overview of the study procedure can be found in the Supplementary Table 1. In addition to the quantitative data, focus group discussions (FGDs) were conducted with the participants of the intervention group. Due to organizational reasons, the intervention was organized in four rounds. In every intervention round, one FGD was conducted 7 months after the onset of the intervention (Figure 1).

### 2.2 Participants

**Figure 2:**
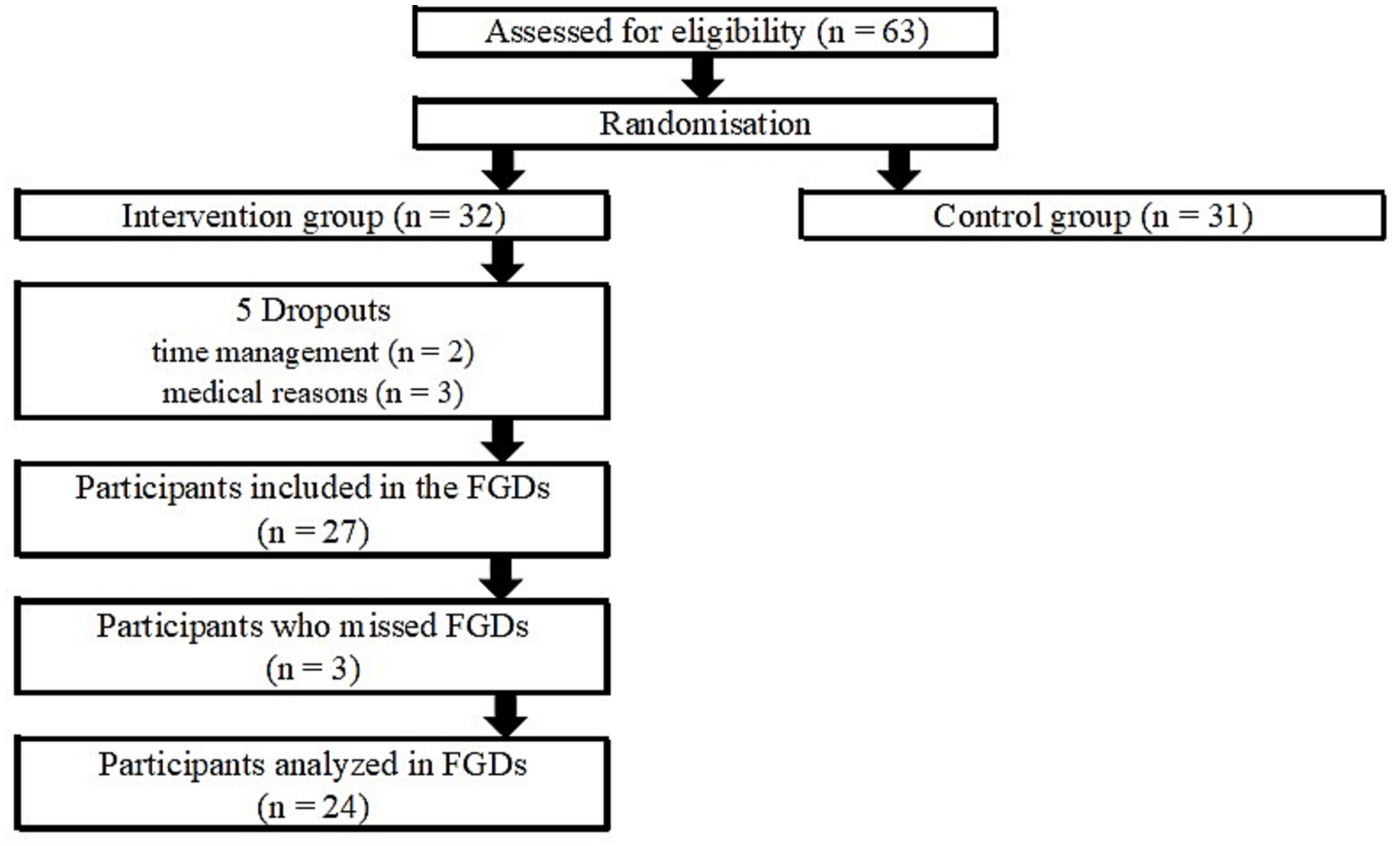
Flow diagram of the NUMOQUA study with focus on the FGDs.

A total of 63 participants took part in the NUMOQUA study. They were recruited from a database of interested patients who had given consent to be contacted from former OA projects of the University of Applied Sciences St. Pölten. Press releases in newspapers and magazines, and networks with self-help groups or specialized clinicians were also used for recruitment. Eligible participants were stratified according to sex and randomized using the Random Allocation Software (46), and allocated either to the intervention (*n* = 32) or the control group (*n* = 31). The FGDs were conducted with all participants in the intervention group. Five participants from the intervention group had to interrupt the study due to personal or medical reasons, and three participants could not take part in the FGDs because of time constraints. The data from 24 study participants in the FGDs were analyzed (Figure 2).

### 2.3 Data collection

In total, four FGDs were held between June 2023 and August 2024 with a total of 24 participants from the intervention group of the NUMOQUA study. The number of participants within each FGD ranged from four to eight. FGDs took place at the University of Applied Sciences St. Pölten and were recorded using Microsoft Teams. FGDs lasted between 75 and 85 min and were led by the two dietitians of the project staff to provide consistency. One of them guided the conversation, while the other one took notes during the FGDs.

The FGDs aimed to provide an insight into the individual perceptions of the participants regarding the “Austrian OA Cuisine.” Topics used to guide the FGDs were determined in advance and summarized in a guideline (Supplementary Table 2). The moderator of the FGDs aimed to initiate an open discussion, while the question guide provided a framework to cover all topics. After a brief introduction and an opening question, participants shared their experiences in following the dietary recommendations in their daily lives, and the barriers and facilitators they perceived when changing their dietary behavior. If necessary, additional questions were asked in order to delve deeper into the topics.

### 2.4 Data analysis

All recordings were transcribed verbatim by one project member and proofread by a second staff member. The names of the focus group participants in the transcripts were anonymized by replacing them with speaker codes, and transcripts were imported into the MAXQDA (Max Qualitative Data Analysis) software (47).

In parallel, *a priori* codebook was developed based on existing literature on factors that shape nutrition and eating behavior, and structured by four influencing spheres (individual, interpersonal and social, environmental, and policy) of the DONE framework (“Determinants of Nutrition and Eating”). The framework provides a systematic, hierarchical structure with a standardized taxonomy for analyzing the determinants of food choices, eating behaviors, and nutrition (48). It ensures a comprehensive coverage of all relevant factors, enhancing transparency and comparability with other studies. The concepts of this socio-ecological framework were uploaded into the coding structure of the MAXQDA software (47). Concepts related to parental determinants were excluded since our study population was above 18 years of age. The data were coded with the 47 *a priori* codes but also using an inductive approach, defining 6 new codes during analysis. The a priori code “food habit” was specified into five sub-categories since it was a dominant code that was discussed by participants with regard to specific preferences, opportunities, changes, routine, and preparation. The *a priori* code “self-regulation” was supplemented by the sub-categories goal-setting and decision making.

A random part of the first transcript (10%) was selected and coded by three coders (SC, UT, and TA), and potential disagreements in coding were discussed afterwards. The same procedure was done with the second transcript. After the two rounds of coding, the coding approach was harmonized, and SC carried out the remaining coding of all four transcripts. During this process, UT and TA discussed ambiguities or decided to develop new codes. Themes were identified by collating data relevant to each code, code group, and the four spheres of influence (individual, interpersonal and social, environmental, and policy) (49). Data saturation did not determine the sample size of the study, but since the same codes and themes reemerged in the course of the analysis, saturation was reached within the dataset (50).

### 2.5 Ethics

The NUMOQUA study was approved by the Ethics Committee of Vienna (Ethics number: EK-22-101-0622; July 22, 2022). Written informed consent was obtained from all participants before the start of the intervention. In addition, informed consent was obtained regarding the recording of the FGDs, which were part of the study participation for the intervention group. All NUMOQUA participants received financial compensation of 100 € and locally produced vegetable oils and nuts after finishing the NUMOQUA study.

## 3 Results

A total of 24 participants (16 women and 8 men) participated in the FGDs. Most participants were between 60 and 69 years old (58.3%), overweight (45.8%), retired (58.3%), married (70.8%), and lived in households with two or more members (75%). Half of the participants lived in urban areas and half in rural areas (51). The sociodemographic characteristics of the participants are summarized in Table 1.

**Table 1:** Socio-demographic characteristics and body mass index of the study participants (*n* = 24).

Characteristic	*N*	(%)
Age		
50–54 years	4	(16.7)
55–59 years	3	(12.5)
60–64 years	6	(25.0)
65–69 years	8	(33.3)
70–74 years	3	(12.5)
Gender		
Female	16	(66.7)
Male	8	(33.3)
Body mass index		
18.5–24.9 kg/m^2^	9	(37.5)
25.0–29.9 kg/m^2^	11	(45.8)
>30 kg/m^2^	4	(16.7)
Education level		
Apprenticeship	3	(12.5)
Vocational school	5	(20.8)
Higher school certificate	9	(37.5)
University	7	(29.2)
Employment status		
Retired	14	(58.3)
Part-time (12–31 h)	3	(12.5)
Full-time (>32 h)	7	(29.2)
Net household income (monthly)		
1.001–2.000 €	3	(12.5)
2.001–3.000 €	5	(20.8)
3.001–4.000 €	6	(25.0)
4.001–5.000 €	2	(8.3)
5.001–6.000 €	3	(12.5)
8.001–9.000 €	1	(4.2)
Not specified	4	(16.7)
Number of persons in household		
1	6	(25.0)
2	11	(45.8)
3	5	(20.8)
4	2	(8.3)
Marital status		
Single	1	(4.2)
Married	17	(70.8)
Divorced	4	(16.7)
Widowed	2	(8.3)
Urban–rural-typology		
Urban area	12	(50.0)
Rural area	12	(50.0)

**Figure 3:**
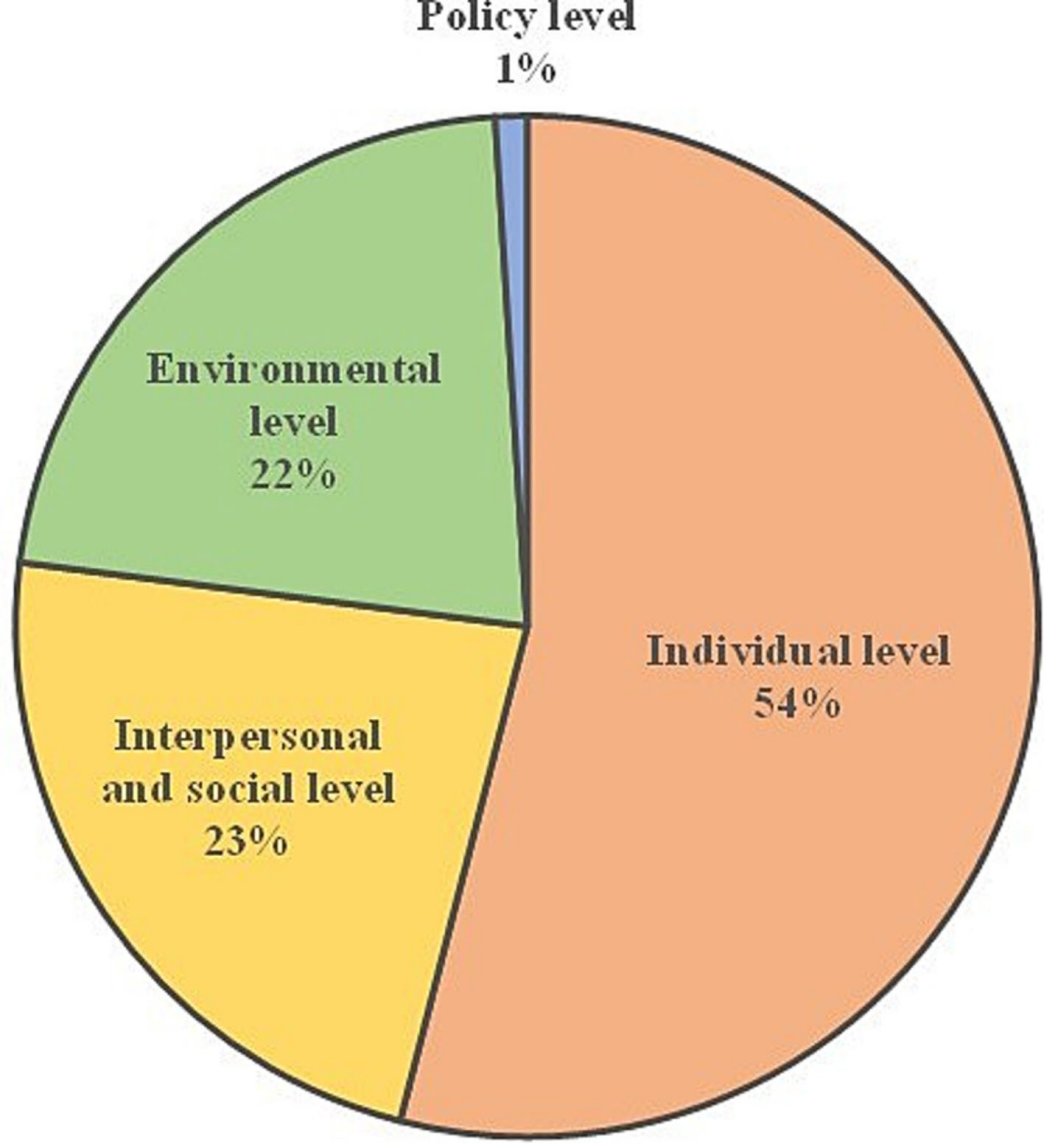
Distribution of the codes according to the influencing spheres of the DONE framework in the FDGs (48).

**Figure 4:**
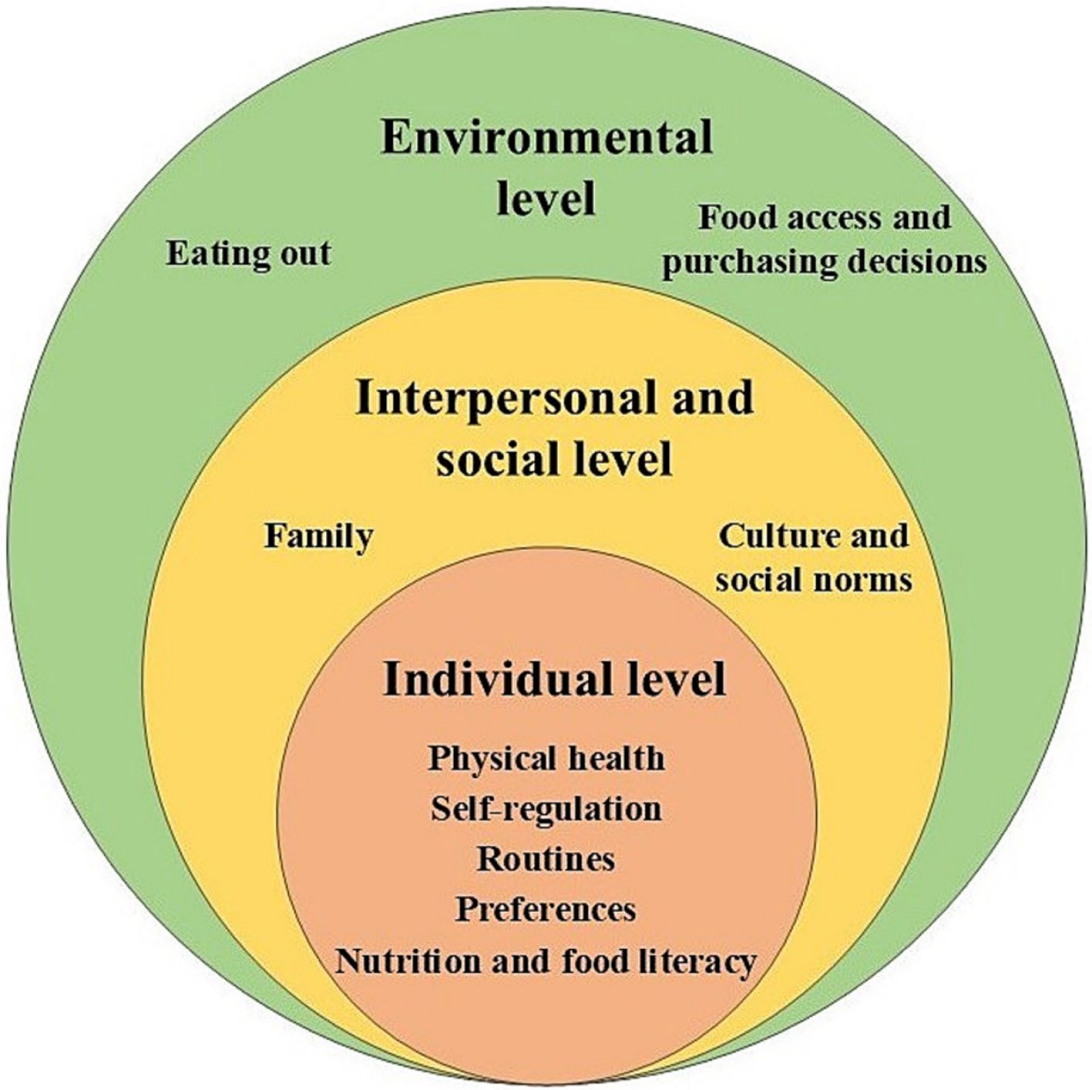
Themes identified in the FGDs based on the DONE framework (48).

The results regarding the barriers and facilitators that the patients experienced in following the recommendations in their daily lives will be presented according to the influencing spheres of the DONE framework (Figure 3). The individual level was most frequently mentioned, with 54% of the codes. Psychological aspects dominated the results at this level, while the biological, demographic, and situational aspects played a minor role in the discussions. An exception was the positive experience relating to physical health (biological aspect), which increased the motivation to follow the program. The interpersonal and social level, and the environmental level were addressed less frequently, with 23 and 22% of the codes, respectively. At the interpersonal and social level, the family played the most important role in influencing the eating decisions of the participants. The environmental level refers to the contextual surroundings that influence dietary behaviors beyond the individual, interpersonal, and social spheres. Most relevant aspects were situations when eating out of the home, and the food availability and accessibility at home or its immediate food environment. Less than 1% of the codes were related to the policy level. As the few individual statements at this level had limited informative value and no themes could be identified, no results are presented here. The following themes were extracted from the participants’ statements in the FGDs (Figure 4) and will be presented in the results together with illustrative quotations. An overview of the themes with extended quotations can be found in the Supplementary Table 3.

### 3.1 Individual level

#### 3.1.1 Physical health

The improvement in physical health during the study was observed as a great motivator to follow the dietary recommendations. Participants described being more aware of their own dietary patterns and how this might influence their general health and quality of life. The benefits in terms of improving physical function and health outcomes related to knee OA even led to previously considered surgical interventions being discarded.

“Also, the awareness has already increased enormously, that you can achieve so much with nutrition” (F, 65).

“I don't need a new knee yet, yes, that almost became an issue a year ago, yes, and this balanced diet I think is the same as with exercise, that has certainly changed the fact that I definitely feel better physically than I did a year ago” (M, 58).

Pain is often the most distressing symptom in knee OA patients’ everyday lives, which was a frequently mentioned aspect that encouraged the participants to follow the recommendations. This was the case both when pain was noticeably reduced and when it persisted.

“That it still hurts me […] that motivates me. Yes, it motivates me to continue” (M, 66).

“It motivates me that my knee doesn’t hurt” (F, 61).

Not only avoiding knee replacement surgery but also reducing or stopping necessary medication was motivating for some participants. This applied both to medication specific for knee OA, but also to other types of medication, such as cholesterol-lowering drugs.

“I don’t need any injections and medications and if that stays the same, it is enough motivation to keep me going” (F, 68).

Participants reported on their other illnesses (gallstones and gastritis), which on the one hand positively influenced their diets, but on the other hand presented sometimes a barrier, e.g., the limited acceptance of various types of fruit in the presence of a cross-allergy.

“Well, because of my gallbladder problems […] it was easy for me to eat really healthy” (M, 66).

#### 3.1.2 Self-regulation

Self-control and goal-setting were the main issues discussed with regard to self-regulation. Setting goals was seen as something useful to focus on concrete dietary changes, even if the goals were not achieved every time. According to the participants, a dietary change should take place step by step, so as not to radically change previous dietary habits, but to slowly and sustainably approach a change. Successfully changing some dietary habits convinced participants that they could stay on track.

“I also have to say that the plans I set myself, I have actually implemented most of them” (M, 69).

“I think it is important to do this steadily and step by step. That you don’t make a big bang right away with a complete changeover” (M, 57).

The participants agreed that a long-term dietary change takes time. Not only to engage with nutrition in a meaningful way, but also to avoid the risk of reverting to old patterns during stressful periods. Participants tried to counteract this by cooking in advance and using quick recipes, but in some cases, stress led to consuming more fast food and ignoring the recommendations.

“Two months ago, I thought to myself: I'm not interested anymore and everything […] was just a lot of stress” (M, 58).

Sometimes the participants were even surprised by what they had achieved and how they had changed their dietary habits.

“Ah, I wouldn’t have thought so [that doing without butter is possible], yes, so it’s a matter of habit after all” (F, 52).

To reach their goals, participants often talked about their discipline and the will to enable changes in their dietary behaviors. Self-control was most frequently mentioned regarding the consumption of sweets and tasty, favorite dishes in the evenings or in stressful phases. Participants were disappointed when they could not withstand their cravings.

“I've already succeeded very much in that I no longer have these food cravings […] that is of course, also the effect of the change in diet” (F, 64).

“I have definitely also reduced the sweets that I used to have in the evening. But it is not completely gone. And sometimes that makes me a bit unsatisfied” (F, 71).

The participants emphasized that a certain freedom in their choice of food was important to them and that they would not accept complete restrictions in their diet. They wanted to be able to make conscious decisions not in line with the recommendations and incorporate expectations, for instance, when eating out, wanting to have their favorite dish, or being sick. They highlighted that enjoyment was an important factor regarding their dietary habits. It was important that they could still treat themselves to their favorite foods without giving them up entirely.

“And if I sometimes eat something unhealthy, well, then I eat it, that’s not so bad, what is important is that you don’t eat it all the time” (F, 58).

“There still has to be room for enjoyment” (M, 57).

#### 3.1.3 Routines

Routines were important for the participants to promote and integrate certain food groups at certain meals (e.g., nuts as a snack or fruits at breakfast). Sometimes, participants included small nudges in their daily routine to push themselves to consume certain foods.

“For example, I eat fruit in the morning. Also nuts and a yoghurt” (F, 65).

“And now, as I already said, the hazelnuts are standing in a glass thingy on my desk” (F, 52).

Enjoying less recommended foods happened as part of routines too, for instance, a piece of cake regularly, or a soft-boiled egg for breakfast on Sundays.

“And once a week, I go to the patisserie” (F, 61).

Additionally, participants noticed that meal structures such as regular meals had positive effects on their wellbeing and food choices throughout the day.

“That's why I now eat three meals a day, because then I'm somehow satisfied and my body doesn't crave anything” (F, 58).

Participants described different forms of support they needed from the dietitian during the intervention period. Repeating the topics of the dietary group training or individual appointments helped the participants to create routines in their daily lives. Especially in stressful phases of their lives, where diet was not a priority, participants felt encouraged and supported by the professional guidance.

“The more often you hear it, the better. This is what learning is about” (F, 53).

“Then the conversations […], you get a bit motivated again and you think again and are reminded of things” (F, 68).

#### 3.1.4 Preferences

For some participants, some dietary patterns were already in line with the dietary recommendations at the beginning of the intervention. Participants described that previous preferences or even cravings for various foods had changed as a result of the intervention.

“And I’ve always liked to eat vegetables. Yes. So, for me this works very well” (F, 69).

“I don't crave meat as much anymore myself” (F, 68).

Sensory characteristics were crucial for personal food choices and were prioritized over the dietary recommendations. Therefore, even clear recommendations did not lead to a change in food choices when participants had an aversion to a food.

“If I don’t even want to smell nut oil or linseed oil today, then yes, I won't eat it either, and then I have a resistance” (F, 52).

The seasons influenced the selection of fruit and vegetables, but also specific beverages and sweets, such as beer and ice cream, in summer.

“Because summer is always dangerous, especially a beer in the evening when it's hot” (M, 58).

#### 3.1.5 Nutrition and food literacy

Although the participants already had a good nutrition knowledge, they perceived an increase in their knowledge, which was considered a relevant factor for following the dietary recommendations in the long-term. As a result, participants felt equipped to make conscious decisions in their daily dietary routine and to act self-efficaciously.

“I’m really looking forward, because I know exactly that I have all the requirements now, yes, in terms of knowing what’s good for me, that is the most important thing that I’ve learned” (M, 58).

For most participants, preparing meals with mostly minimally processed food was something they had already done before. As a result, the preparation of the “Austrian OA Cuisine” was also seen as something feasible in everyday life, although a special cooking course would have been considered helpful by some participants, especially for more complex dishes or unfamiliar foods. The recipe book provided by the NUMOQUA study was seen as a source of motivation and reference. Most participants used the OA cuisine recipes but also used them as an inspiration to adapt what they usually cooked (e.g., increasing whole grain content, decreasing sugar content, replacing fats, or using low-fat products).

“But otherwise, in the beginning […], I stuck to the recipes. So. Now I have them in my head, though” (F, 61).

“Yes, that [note: recipe book] is already very helpful, but I would prefer to take a cooking workshop sometime” (F, 58).

“And I use rapeseed oil now instead of butter. That's the difference, but I do need something sweet” (F, 65).

Although participants already had a cooking routine before the study, they described increasingly preferring home-made meals for different reasons. They argued that cooking and baking gave them more control over the ingredients, for instance, regarding fat sources or whole-grains, and the quality of the products used, regarding the origin of the food or organic certificates.

“Yes, well, you don't know that [note: if organic food is used] at the restaurant, so I prefer to cook at home” (F, 58).

“I like to eat an apple pie or an apple strudel or things like that, I don't go without them […] I just make everything myself now, I don't buy pastries” (F, 52).

The majority agreed that there was no additional time needed to prepare and purchase food for a PBD. Only a few participants stated that they needed more time to include and prepare new foods, such as pulses. In contrast, cooking vegetarian dishes was perceived as faster than cooking meat.

“But I don't think it's any more work now [note: preparation of the OA cuisine compared to other diet]” (M, 60).

### 3.2 Interpersonal and social level

#### 3.2.1 Family

Especially female participants were used to adapt their food choices to the family members and prioritize the wishes of others over their own. As a result, certain food groups were not on the menu at all.

“Well, it's not that I don't like it [note: pulses]. It's just that I've never cooked it because my husband doesn't like it and that's why it wasn’t part of my diet at all” (F, 53).

Sometimes compromises were made to ensure that family members accepted the meal while also partially satisfying one’s own needs, e.g., the partial use of whole meal flour. In some cases, where family members requested meat, only part of the meal was eaten by the study participants.

“No, I don't cook separately, if there is meat, then I only eat side dishes, for example I only eat the potatoes with vegetables” (F, 53).

In other cases, partners were supportive in the dietary transition, especially when the partner was the one who usually cooked at home.

“I'm not the main chef, yes, but my wife […] is totally on board now, yeah, I have to say, that's the good thing, yes” (M, 58).

Another important factor was related to which partner worked or who spent more time at home. The partner who stayed at home due to early retirement or unemployment played a decisive role in the meal preparation.

“My partner is a great support at the moment, she has unfortunately lost her job, but that has given her a lot of time and she now cooks all the food” (M, 60).

If the participants were responsible for preparing food and family members accepted the adapted recipes, it helped them to satisfy everyone in the household. When family members already showed health awareness and had plant-based eating habits, participants perceived this as supportive for following a PBD.

“But she [note: wife] has actually always had a very healthy diet from the beginning” (M, 57).

The participants perceived generational differences, whereby the dietary patterns of the older generation were seen as so entrenched that they were taken for granted, and no attempts were made to change them.

“My father, who is 88 years old, is a true carnivore. If he's there, then you can't cook without meat” (M, 58).

Participants also reported an impact on dietary behaviors through a change in the household composition. When children moved out or the partner passed away, dietary compromises with regard to food choices were obsolete.

“I didn't find it difficult to make the change because it was only my husband who always wanted meat and now, sadly, he has died. And that meant I could cook what I wanted and it was easier” (F, 69).

#### 3.2.2 Cultural and social norms

Religious celebrations, cultural occasions, and community dietary habits often interfere with dietary recommendations. Traditional dietary habits are so ingrained that they were not changed due to the intervention.

“My three boys […] and I went to the stadium to watch Austria versus Sweden [note: soccer match], and before that we went to the Schweizerhaus [note: traditional Viennese restaurant famous for pork dishes] and had a freshly tapped Budweiser, which was a must” (M, 58).

Even if participants were willing to change, there were no appropriate choices at the different festivities.

“The only thing I always have to worry about is playing my organ [note: at funerals] […] and then I'm always in church and there's always a meal afterwards […] and then there's Wiener schnitzel, dessert, coffee, and roast pork with all the trimmings” (M, 60).

“And also at the wedding, for example, the only thing [note: vegetarian] was the baked mushrooms” (M, 60).

### 3.3 Environmental level

#### 3.3.1 Eating out

Eating out was seen as both a resource and a barrier to following the dietary recommendations. Eating out allowed choosing vegetarian or fish options in restaurants, which were limited in range, or unattractive, and sometimes contained a lot of fat.

“Not eating meat isn't really a problem at all now, it's only when you're, like, invited to a restaurant or something […] that there's nothing really vegetarian on the menu” (M, 60).

Following the dietary recommendations during holidays was dependent on the destination and type of holiday. Participants described consuming more fish in seaside holiday resorts, but also that a healthy selection of food was more expensive. They developed strategies to promote healthy food choices when there was a wide range of dishes (e.g., buffets).

“But I don't think that's a problem, because on holidays, for example, I had fish almost every day” (F, 68).

“Choosing from the buffet, starting with the salad and vegetable side, and then moving on to the meat. So more or less against the flow, so there wasn't so much room on the plate [note: for meat]” (M, 66).

In situations where the participants had no influence on what food was offered to them by others (e.g., at invitations), they dealt with them differently. In some cases, these opportunities were seen as an obstacle, in other cases as a relief.

“I noticed that it [note: following the diet] works less well, […] e.g., at an invitation to a barbecue” (M, 66).

“You’re happy that you don’t have to cook by yourself [note: at invitations] and eat what is on the table” (F, 61).

#### 3.3.2 Food access and purchasing decisions

Some participants emphasized that shopping was the crucial factor for them when changing their diet. Through shopping, they could limit what they had available at home and avoid eating unhealthy food or possibly having to throw it away.

“For me, changing my diet definitely starts with shopping. If I don't have the stuff at home, then I can do without it quite easily” (M, 66).

Participants chose food based on a wide range of values, which were prioritized differently when shopping for food. They tried to buy from small local suppliers, markets, or butchers and took organic, seasonal food and animal welfare into account, but this was not always feasible. Shops had to be close to their homes or on the way to or from work. Additionally, access to some foods, such as nuts, was limited, as they were not locally available.

“We probably have, I don't know, regional shops somewhere, but not in Vienna where I work, so I just go to Billa [note: supermarket]” (F, 58).

“The main focus is always locally sourced, the second focus is organic” (F, 52).

High-quality foods, such as organic products and wholemeal products, or sourcing directly from the producer, were associated with higher costs. At the same time, participants thought that these high costs were balanced out by a healthy diet, where savings can be made by avoiding other food groups.

“And basically, if you buy higher-quality food, then it's more expensive” (M, 66).

As half of the participants lived in rural areas, they often owned a garden, which influenced their food choices. Home gardens contributed to the supply of vegetables, fruits, and nuts, and were consciously used to change the food choices.

“I mean, with the pulses, I never used to cook them like that. I’m growing a lot of them in the garden this year, so I’ll definitely cook them” (F, 68).

Using their own gardens as a source of food was aligned with the seasons, which indirectly influenced the dietary behaviors of the participants. They also used different methods of food storage, like freezing, to make food such as berries and vegetables available for longer periods.

“I cook differently in summer than in winter, so in summer I cook things from the raised bed and in winter I cook these lentils and beans and all that” (F, 62).

The participants described an inner conflict at the beginning of the intervention, as they did not want to throw away foods that were not recommended. As a result, these foods were used up, which delayed the transition to the recommended “Austrian OA cuisine.”

“I also did very well during the changeover – at least after I had used up everything that was still there” (F, 61).

## 4 Discussion

This study aimed to identify perceived barriers and facilitators for adopting the dietary recommendations of a therapeutic PBD in patients with knee OA. Participants received comprehensive group training and individual counseling from a dietitian to help them incorporate the diet into their daily lives. The goal was to ensure an adequate nutrient intake, particularly focusing on the fatty acid pattern, sufficient omega-3 fatty acid intake, reducing animal-based foods (especially meat), and consuming an adequate amount of fiber and phytonutrients through plant-based foods. The main barriers for adopting the dietary recommendations were the availability of options when eating out, traditional dietary habits during celebrations and cultural occasions, and prioritizing the needs of family members. However, family members were also mentioned as supportive, alongside goal-setting, creating routines, motivation due to improved physical health, and an individual approach in the dietary intervention. The results of the FGDs reflect the various influencing factors, with aspects at the individual level playing the greatest role in following the recommendations. Factors on the interpersonal and social level, as well as the environmental level, were also very important for the participants. Themes from all three areas were perceived as both barriers and facilitators. Influencing factors related to the political level were hardly mentioned.

At the individual level, the participants were motivated to adhere to the dietary recommendations due to the perceived improvement in their physical health, especially the reduction of pain, even though they were unable to determine whether the improvement was due to the dietary change or the concurrent training program GLA:D^®^. Participants’ statements confirm previous study findings that OA symptoms, including pain, motivate patients to follow a healthy diet (29, 52). Since pain is a major barrier to exercise (53), the pain relief could subsequently lead to an increase in physical activity (54), which is strongly recommended as a first-line treatment in the management of knee OA (39). These physically perceived experiences can help patients with knee OA recognize that conservative disease management not only bridges the gap until surgery is necessary, as indicated by the results of a qualitative study (55). Moreover, it represents a justified option for improving quality of life, slowing disease progression, and potentially avoiding surgery.

In order to make recommended dietary changes, participants needed time, individual solutions, and flexibility. The effect of the duration or mode of delivery of a dietary intervention on dietary behavior in non-communicable diseases remains unclear (56), but a systematic review found that dietary interventions in older adults were more effective when performed in group sessions and with a follow-up period of more than 6 months (57). The long duration of the study made it possible to guide the participant through different stages of change according to the transtheoretical model of behavior change (58). The dietary intervention of the NUMOQUA study addressed the stages of preparation (intend to take action), action (modify the behavior), and maintenance (consolidate the gains), by implementing different behavior change techniques like self-management, self-motivation, problem-solving, and goal-setting (59). As described in the literature (58), participants especially mentioned goal-setting and adapting by small steps to the recommendations as highly important for the dietary change. The regular meetings with the dietitian in this study made it possible to review the latest experiences, to provide support when dietary changes were not progressing smoothly, and to encourage patients that shifting the diet is not a linear process (58, 60). Routines seemed to be decisive for the participants when it came to making a dietary change, not just in the short term, but as a long-term integration into everyday life. These results further support the idea that habit formation is a key determinant in dietary behavior change. Previous studies report that habit formation can be effective—both linking the dietary behavior to a daily routine and a specific time—and eating decisions are subsequently made automatically and unconsciously (61, 62). Participants of our study requested a lot of flexibility due to their varying needs. These included the absence of strict dietary requirements, the consideration of individual taste preferences, and no restrictions on different foods. The literature on flexible control of eating behavior, the development of cravings, and the effect on dietary outcomes is not consistent. Flexible dietary control strategies were positively associated with diet success, weight loss maintenance, and psychological wellbeing, and indicated that rigid control strategies lead to self-regulatory failure in dietary behavior (63, 64). However, a more recent review argues that this may only apply in the short-term. In the long-term, food deprivation can facilitate the elimination of conditioned food craving responses (65). This finding is also supported by participants’ reports, some of whom stated that their cravings for certain foods completely disappeared.

At the interpersonal and social level, family was the most prominent theme in the discussions—experienced both as a barrier and a supportive factor. In most cases, the decisive person was the partner or spouse, but other family members, such as parents, children, or grandchildren, also influenced the dietary behavior of the participants. Family support, health awareness, and plant-based dietary habits of family members were perceived as important factors that facilitate adherence to a therapeutic PBD. Lund and Halkier (66) showed in a Danish study that the decision to eat less meat increased with the number of personal contacts, particularly close family members and friends, who ate small amounts of meat or were vegetarians or vegans. However, it seems that traditional gender roles were highly relevant in the participants’ family structures, which may be due to the age group of the people studied. Men consistently reported strong support from their partners and wives, who also predominantly prepared the food and shopped for groceries. Women, on the other hand, struggled more often with reconciling their own desires with those of household members. Similar to what another study in Denmark found (67), maintaining harmony and cohesion at family dinners seemed also important to our female participants. The husbands or partners were perceived as major barriers to women trying to consume less meat, confirming previous results from FGDs with women who are living with their families (68). Men, especially in the “baby boomer” cohort, have a lower willingness to transition to more PBD than women (69), and meat consumption is strongly associated with masculinity (70). Women adapted to the family preferences partially by cooking different meals or more often by omitting certain meal components, especially meat. If no other sources of protein are consumed instead, this can worsen the nutritional content of the meal and the protein intake, which could be particularly problematic for musculoskeletal health in women at this age (71). Therefore, plant-based protein sources, such as legumes, nuts, and whole grains, are important for ensuring an adequate protein intake in plant-based diets of older women (72).

In contrast to earlier studies, which show that living alone negatively impacts the cooking and dietary habits of older people (73, 74), participants mentioned living alone provided an opportunity to cook healthy meals since they no longer had to consider the preferences of other household members. As this was consistently expressed by women, this may be due to the fact that single women consume generally healthier diets than single men (73). In addition to the family, the participants also perceived cultural traditions and religious festivities as barriers. High-fat meat dishes were often served at these events, while plant-based options were lacking. Preparing and consuming meat is strongly linked to cultural traditions and norms in Western societies, associated with prosperity and a high social status (75), and is therefore offered at celebrations of special occasions. Moreover, at invitations in private settings, hosts are increasingly offering meat, even if they are flexitarians themselves, responding to perceived social expectations (76). Consequently, meat consumption in Austria remains very high at 58 kg per capita (77), far exceeding the recommendations of the national food-based dietary guidelines (38).

The environmental factors mainly concerned the availability, access, and attractiveness of plant-based options when eating out or purchasing food. Eating out in restaurants or on holidays was perceived as a barrier as well as a facilitator. Participants considered these occasions as opportunities to eat food that they cooked less at home, both the recommended choice (e.g., fish) and the less recommended one (e.g., typical Austrian meat-dishes like “Wiener Schnitzel”). The latter confirms previous data that eating out in restaurants can increase the likelihood of consuming meat compared to eating at home (76, 78). Literature indicates that taste is the most important criteria when eating in restaurants, and meat-eaters are concerned about the taste of plant-based dishes. For this reason, meat dishes at restaurants are considered “more worthy” and are associated with pleasure and treating yourself (76, 79). On the other hand, participants perceived limited or unattractive plant-based options in restaurants as a barrier. A previous review states that eating out frequently is associated with poorer diet quality and lower intake of vegetables (80), but a German survey showed that there was a higher willingness to shift to a PBD if supermarkets, restaurants, and workplace cafeterias offered more plant-based options (69). Food environments, therefore, influence people’s capacities to make healthy or sustainable choices (81) and have been associated with poor diet quality and obesity (82, 83).

Purchasing food was an important factor in making long-term dietary changes. This is in accordance with earlier observations, which showed that food purchases reflect the diet quality of food shoppers (84). Participants tried to avoid food waste by using up the food items at home, even if they were not recommended, before switching to the recommended ones (e.g., the choice of vegetable oils). This may be due to the good food management skills in this age group, like knowing about food storage, reusing leftovers, and consequently reducing food waste (85). For the dietary intervention, this meant that following the recommendations might have been delayed by the patients. The home garden was a valuable resource for participants regarding the availability of fresh fruit and vegetables, and confirms previous positive associations between home gardening and fruit and vegetable intake (86). Although the four FGDs took place at different seasons of the year, home gardening was mentioned in all four focus groups.

Representing industry and government regulations and campaigns, the policy level took up little space in the FGDs. The few comments in this category expressed a wish for increased cooperation between the various stakeholders in the Austrian healthcare system and investment in chronic disease prevention. The policy level was not specifically addressed in the question guideline of the FGDs, but the few comments could also reflect that participants experienced the individual choices and their immediate environment more strongly than the nutrition policy actions (87). However, policy actions are needed to improve the availability, access, and affordability of sustainable and healthy diets for all. Availability and visibility of plant-based meals in restaurants or canteens, especially in rural settings, where the study participants lived, needs to be increased, for instance, through more prominent displays and labeling in menus or in canteens (88, 89).

Findings of this study can be used to improve patient-centered communication and enhance the effectiveness of dietary interventions. The NUMOQUA study invested more time in nutrition education than a dietitian has in the daily clinical practice, where patients often do not receive information or motivation from health professionals to consider conservative treatments, such as dietary interventions (55). Providing patients with information about the dietary potential in the disease management of knee OA could counteract the fact that patients instead use dietary supplements, or turn to other resources (e.g., websites and social media) (90, 91). Our data reinforce previous findings that self-monitoring, social support, more information from various health professionals, and guidance are essential for patients in the disease management of OA (92, 93). The aspects of “information” and “guidance” were well covered in this study, which helped participants to overcome two known main barriers for adopting a PBD (27): (1) concerns about meeting nutritional requirements and (2) a lack of knowledge about what to eat in a PBD were not relevant for participants in this study. In a European survey, participants from Germany and Austria scored barriers for shifting to a PBD lower than in other countries. This suggests a readier market for a diet shift in German-speaking countries (94).

### 4.1 Research and program implications

Results of this study highlight the potential of promoting therapeutic PBDs in individual dietary interventions in order to promote sustainability. Our data underline the importance of goal-setting, creating routines, and the individual approach in dietary interventions. Dietitians should use techniques to support these aspects and deliver individual, personalized counseling to enable patients to make conscious decisions in their food choices, without imposing strict dietary restrictions. Barriers related to the family context and eating out also need to be addressed in dietary counseling. Interventions may require more intensive involvement of partners and family members to further enhance adherence to dietary recommendations in everyday family life. While the lack of options when eating out is beyond the control of patients and dietitians, they can still discuss how the patient can manage this in their daily life. Additionally, dietitians should be aware that patients with OA have different preferences regarding the focus of dietary interventions and address these topics even in the absence of empirical support (90).

Future research could identify solutions to overcome the perceived barriers related to the influence of family members, particularly male partners. Another research area concerns the limited availability of plant-based meals in restaurants located in rural areas in Austria. Future studies should further assess the influence of limited availability on dietary behavior and identify strategies to increase the availability of plant-based meals in restaurants in rural areas. The effective integration of dietary recommendations into the standardized disease management of OA should also be further investigated.

### 4.2 Strengths and limitations

While other qualitative nutritional studies with knee OA patients focus on weight loss approaches (95, 96) or the process evaluation of lifestyle programs (93), this article presents findings on the barriers and facilitators experienced by knee OA patients following a therapeutic PBD. The content of the FGDs was analyzed along an established theoretical model, which allowed systematically analyzing the results. The heterogeneous composition of the FGDs in terms of gender, living area, income, and level of education provides a broad insight into the topic. Since the participants were in the middle of the intervention, they could recall their immediate experiences instead of relying on memory, but it remains unclear whether the experiences would differ in the long term. The participants in each FGD already knew each other as they had taken part in group training sessions together as part of the intervention. Consequently, there was a very familiar and pleasant atmosphere at the FGDs, where participants talked openly. However, it cannot be ruled out that socially desirable statements were made about their own dietary behavior.

One limitation of the study is that the findings are not representative for all patients with knee OA due to the age of the study participants, the setting of the study in one region of Austria, the assumed above-average motivation of the participants, and the intensity of the interdisciplinary lifestyle intervention. Although it was communicated clearly and repeatedly that the conversation in the FGDs focused on dietary behavior, it is possible that some of the participants’ statements (e.g., about motivation) were also influenced by the accompanying training program.

## 5 Conclusion

Promoting a therapeutic PBD in patients with knee OA is crucial to improve their health while contributing to planetary health. Reducing overweight and following an anti-inflammatory PBD positively influences disease progression and improves risk factors for related comorbidities, such as cardiovascular diseases and diabetes mellitus. Results of this study show that following a therapeutic PBD in patients with knee OA is influenced by a variety of individual, interpersonal and social, as well as environmental factors. Data could strengthen future dietary interventions to be more holistic in order to enable long-term behavioral change and also increase awareness of sustainable diets. Data suggest that measures regarding the nutritional environment are at least as important as those at the individual level. The results not only contribute to a better disease management of OA, but also provide valuable information for the promotion of PBDs in other non-communicable diseases, particularly cardiovascular diseases, type 2 diabetes, and obesity.

## Data Availability

The datasets presented in this article are not readily available because the data that has been used is confidential. Requests to access the datasets should be directed to sabine.chmelar@ustp.at.
