# Supplementary material for: Barriers and facilitators for consuming a plant-based diet in patients with knee osteoarthritis: a qualitative study
Source: Front Nutr. 2026 Mar 13;13:1743219. doi: 10.3389/fnut.2026.1743219 (PMC13021465; doi:10.3389/fnut.2026.1743219)
Supplement: Supplementary file 1 [file Table_1.DOCX]

**Supplementary file A**

**Overview of the study design of the NUMOQUA study**


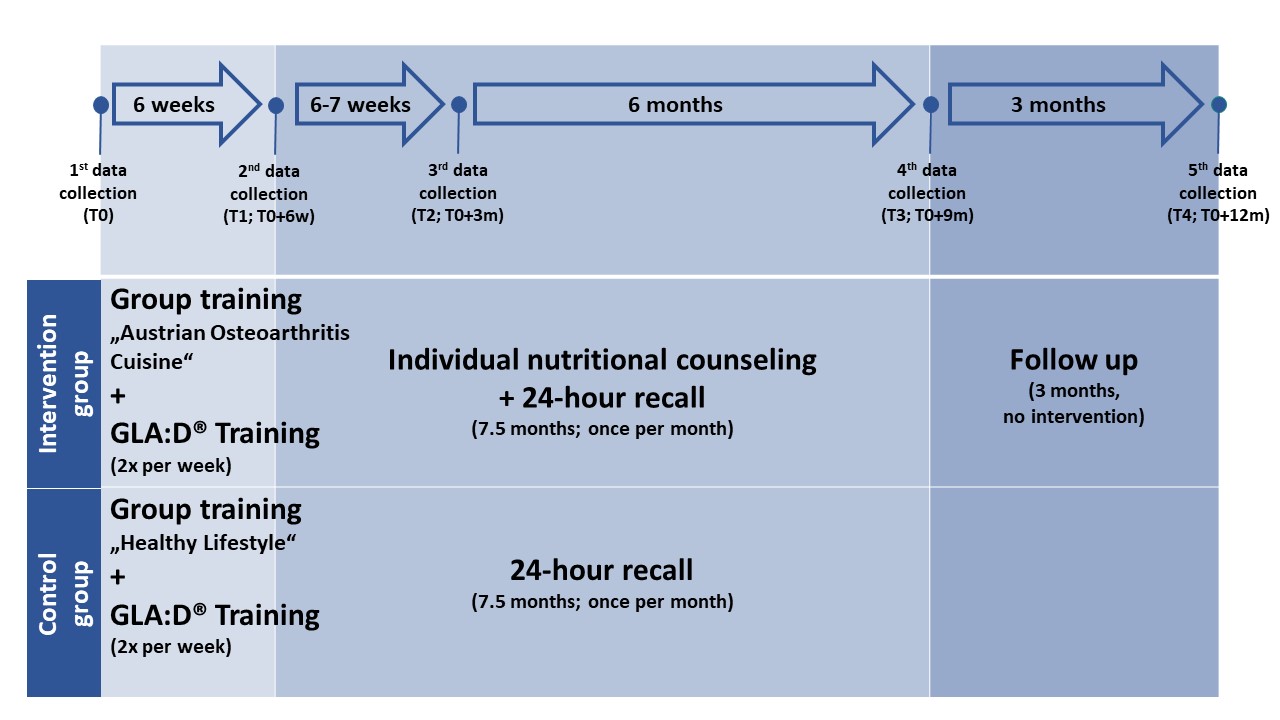


Data collection included:

- Patient-reported outcomes
- Blood-based biomarkers
- Clinical Data
- Behavioral parameters
- Anthropometric parameters
- Qualitative data (focus groups)

The detailed study protocol can be found here:

Höld, E., Chmelar, S., Aubram, T., Leitner, G., Nehrer, S., Neubauer, O., Wagner, K.-H., & Wondrasch, B. (2024). Nutrition and movement to improve quality of life in patients with knee osteoarthritis: The NUMOQUA study protocol for a randomised controlled trial. *Trials*, *25*(1), 245. <https://doi.org/10.1186/s13063-024-08048-2>
