# Supplementary material for: Barriers and facilitators for consuming a plant-based diet in patients with knee osteoarthritis: a qualitative study
Source: Front Nutr. 2026 Mar 13;13:1743219. doi: 10.3389/fnut.2026.1743219 (PMC13021465; doi:10.3389/fnut.2026.1743219)
Supplement: Supplementary file 2 [file Table_2.DOCX]

**Supplementary file B**

**Question guide for the focus group discussions (FGDs)**

Two members of the project staff guide the FGDs: one is the moderator, one takes notes.

# Introduction

The moderator gives a brief introduction about the purpose and formality of the FGDs. Participants are encouraged to share their experiences openly and respectfully, without rating the statements of the others. After participants give consent for recording with Microsoft Teams, the recording starts. Water, fruits and nuts are provided during the FGDs.

# Warm-up

The discussion starts with a warm-up question to stimulate the exchange of experiences and involve everyone.

Question: “What would you tell a friend about the NUMOQUA study?”

# Theme 1: Acceptance of the dietary recommendations

Question: “What are your experiences following the dietary recommendations?”

Follow-up questions (examples):

“Which new experiences did you make?”

“What went well?”

“What was difficult for you?”

# Theme 2: Barriers following the dietary recommendations

Question: “Which barriers did you perceive in changing your dietary behavior?”

# Theme 3: Facilitators following the dietary recommendations

Question: “Who or what helps you following the dietary recommendations?”

Follow up questions (examples) for Theme 2 and 3: additional questions regarding e.g., the role of the family, society, different eating situations, cooking, purchasing food, …

“Could you give us an example?”

“Would you like to add something?”

“Could you explain this further?”

# Closing

The moderator ends the session by asking participants about their overall experiences with the project

Question: “What motivates you to maintain lifestyle changes?”

“What was most important for you in the study setting?”

“What would you change about the project if you were responsible for it?”

The moderator asks if anybody wants to add something. The two project team members give the participants an outlook on the further procedure of the study. Participants are thanked for their time, contribution, and personal insights.
