# Supplementary material for: Barriers and facilitators for consuming a plant-based diet in patients with knee osteoarthritis: a qualitative study
Source: Front Nutr. 2026 Mar 13;13:1743219. doi: 10.3389/fnut.2026.1743219 (PMC13021465; doi:10.3389/fnut.2026.1743219)
Supplement: Supplementary file 3 [file Table_3.DOCX]

**Supplementary file C**

**Table of themes and illustrative quotations**

| **INDIVIDUAL LEVEL** |
| --- |
| **Theme: Physical Health** |
| *“I have found that it does me good. So, both physiotherapy and the nutrition-topic, and therefore I’m sticking with it.”* (M, 66)  *“I don't need a new knee yet, yes, that almost became an issue a year ago, yes, and this balanced diet I think is the same as with exercise, that has certainly changed the fact that I definitely feel better physically than I did a year ago.”* (M, 58)  *“That it still hurts me [...] that motivates me. Yes, it motivates me to continue.”* (M, 66)  *“It motivates me that my knee doesn’t hurt.”* (F, 61)  *“I'm motivated by the fact that my knee doesn't hurt and that I've lost a bit of weight.”* (F, 61)  *“I don’t need any injections and medications and if that stays the same, it is enough motivation to keep me going.”* (F, 68)  *“I had a winter without knee pain this year. I got through it without medication.”* (F, 73)  *“My big goal was that I wouldn't have to take my cholesterol pills, and I'm not taking them yet.”* (F, 52)  *„Well, because of my gallbladder problems […] it was easy for me to eat really healthy.”* (M, 66)  *“And the butter is the fat issue [...] that has to do with the fact that I always have problems with my stomach. And that also ran in parallel.”* (M, 66) *“And I have my problems with fruit. Because of my allergy. Stone fruit is always a problem [...] and there are cross-allergies.”*(M, 66) |
| **Theme: Self-regulation** |
| *“I also have to say that the plans I set myself, I have actually implemented most of them.”* (M, 69)  *“I think it is important to do this steadily and step by step. That you don’t make a big bang right away with a complete changeover.”* (M, 57)  *“Or if I give up everything that I used to like to eat or that I would like to eat again, it frustrates me and then I fall back into something completely different.”* (F, 73)  *“Two months ago, I thought to myself: I'm not interested anymore and everything [...] was just a lot of stress.“* (M, 58)  *“And now that I'm going through a stressful phase, I feel like eating sweets, but as I said, I'm relatively consistent in that I don't give in all the time.“* (M, 60)  *“Ah, I wouldn’t have thought so [that doing without butter is possible], yes, so it’s a matter of habit after all.”* (F, 52)  *“I would not have been believed that in the past. I couldn’t have imagined that in the past [reduction of sweets consumption].”* (M, 66)  *“And yes, it was rather the case that I sometimes didn’t achieve my goals […] occasionally there is something of a deviation visible.”* (M, 69)  *“That's [note: not buying sweets] the discipline I impose on myself.”* (F, 62)  *“I've already succeeded very much in that I no longer have these food cravings [...] that is of course also the effect of the change in diet.”* (F, 64)  *“I have definitely also reduced the sweets that I used to have in the evening. But it is not completely gone. And sometimes that makes me a bit unsatisfied.”* (F, 71)  *“And if I sometimes eat something unhealthy, well, then I eat it, that’s not so bad, what is important is that you don’t eat it all the time.”* (F, 58)  *“I think about the eighty-twenty rule. Eighty percent of my nutrition is like the arthrosis cuisine intends it, and sometimes I make exceptions and that’s that.”* (F, 58)  *“But I also eat roast pork or cordon bleu. I don’t want to miss out on that.”* (F, 61)  *“There still has to be room for enjoyment.”* (M, 57)  *“There has to be a little bit of joy as well.”* (F, 52) |
| **Theme: Routines** |
| *“For example, I eat fruit in the morning. Also, nuts and a yoghurt.“* (F, 65)  *“And now, as I already said, the hazelnuts are standing in a glass thingy on my desk.”* (F, 52)  *“And once a week, I go to the patisserie.”* (F, 61)  *“A soft-boiled egg on Saturday. No more than that.”* (M, 60)  *“That's why I now eat three meals a day, because then I'm somehow satisfied and my body doesn't crave anything.”* (F, 58)  *“And I really try not to eat after at least 5pm [...] you then have more energy the next day.”* (M, 66)  *“Also, in the afternoon [...], I used to have cravings for chocolate and stuff like that. But if I eat a balanced lunch now, then I don’t have that.“* (F, 58)  *“The more often you hear it, the better. This is what learning is about.“* (F,53)  *“It's also important that you simply talk about it, yes [...] that you talk long enough for it to really sink in.”* (F, 53)  *“Then the conversations [...], you get a bit motivated again and you think again and are reminded of things.”* (F, 68)  *“So, for me it [the regular conversations] was the key, because otherwise you are so far away after two months, then phew, then you easily let it slide I think.”* (M, 58)  *“Well, I would not want to return to the old routines. Not now that I have come this far.”* (M, 57) |
| **Theme: Preferences** |
| *“And I’ve always liked to eat vegetables. Yes. So, for me this works very well.”* (F, 69)  *“Yes, and like already said, I never much liked to eat meat anyway.”* (F, 69)  *“I don't crave meat as much anymore myself.”* (F, 68)  *“Because before, I didn’t eat whole meal products at all, I didn’t need them, and now I like eating them, I like the taste and well, it fits.”* (F, 69)  *“If I don’t even want to smell nut oil or linseed oil today, then yes, I won't eat it either, and then I have a resistance.“* (F, 52)  *“Because summer is always dangerous, especially a beer in the evening when it's hot.“* (M, 58) |
| **Theme: Nutrition and food literacy** |
| *“I’m really looking forward, because I know exactly that I have all the requirements now, yes, in terms of knowing what’s good for me, that is the most important thing that I’ve learned.”* (M, 58)  *“I mean, I did know that I wasn’t leading a healthy lifestyle or that I didn’t follow a healthy diet, but I didn’t do any research beforehand, so this has really helped me a lot and also helped with the changes.”* (M, 60)  *“The most important topic for me, I think, was nutrition, being aware of what I eat in the first place. I hadn’t thought about that before.”* (M, 58)  *“Yes, that [note: recipe book] is already very helpful, but I would prefer to take a cooking workshop sometime.”* (F, 58)  *“Sure, I can do that [cook simple meals], I do it myself all the time, yes, but very complex meals, no, I can’t do them.”* (M, 60)  *“Yes, well, you don't know that [note: if organic food is used] at the restaurant, so I prefer to cook at home.”* (F, 58)  *“I like to eat an apple pie or an apple strudel or things like that, I don't go without them [...] I just make everything myself now, I don't buy pastries.”* (F, 52)  *“And I use rapeseed oil now instead of butter. That's the difference, but I do need something sweet.”* (F, 65)  *“I've also switched to wholemeal flour and I make my own bread.”* (F, 69)  *“So, I've leafed through the cooking book myself [...] I've read it once, but in the end [...] I don't do it then.”* (F, 52)  *“But I don't think it's any more work now [preparation of the OA cuisine compared to other diet].”* (M, 60)  *“What I used to cook for two hours, because if there's meat, well, you know [...] and now with the vegetables, it's done in no time at all.“* (F, 69)  *“So especially when it comes to integrating pulses [...] I have to [...] first of all find a recipe [...] and then it just takes me a while to get to grips with it.“* (F, 71)  *“But otherwise, in the beginning [...], I stuck to the recipes. So. Now I have them in my head, though.”* (F, 61) |
| **INTERPERSONAL AND SOCIAL LEVEL** |
| **Theme: Family** |
| *“Well, it's not that I don't like it [note: pulses]. It's just that I've never cooked it because my husband doesn't like it and that's why it wasn’t part of my diet at all.”* (F, 53)  *“When the granddaughters visit me, we have rice pudding and pancakes”* (F, 73)  *“No, I don't cook separately, if there is meat, then I only eat side dishes, for example I only eat the potatoes with vegetables.“* (F, 53)  *“The compromise when it comes to pastry is that I just replace a third with wholemeal flour because otherwise mine [note: husband] won't eat it.”* (F, 52)  *“I'm not the main chef, yes, but my wife [...] is totally on board now, yeah, I have to say, that's the good thing, yes.”* (M, 58)  *“My partner is a great support at the moment, she has unfortunately lost her job, but that has given her a lot of time and she now cooks all the food.”* (M, 60)  *“But she [note: wife] has actually always had a very healthy diet from the beginning.”* (M, 57)  *“Although my children are vegan and I didn't have to tell them that much because they do it better than I do.”* (F, 61)  *“My father, who is 88 years old, is a true carnivore. If he's there, then you can't cook without meat.“* (M, 58)  *“You grow up with that and my father-in-law is the same, so if there's no meat at snack time [...] it comes from society.“* (F, 54)  *“I didn't find it difficult to make the change because it was only my husband who always wanted meat and now, sadly, he has died. And that meant I could cook what I wanted and it was easier.“* (F, 69)  *“When the children were still at home, the fridge was always full of all sorts of things anyway. Now that the children are no longer at home [...], I don't buy as much meat or anything else just for myself.“* (F, 54) |
| **Theme: Cultural and social norms** |
| *“My three boys […] and I went to the stadium to watch Austria versus Sweden [note: soccer match], and before that we went to the Schweizerhaus [note: traditional Viennese restaurant famous for pork dishes] and had a freshly tapped Budweiser, which was a must.”* (M, 58)  *“The only thing I always have to worry about is playing my organ […] and then I'm always in church and there's always a meal afterwards […] and then there's Wiener schnitzel, dessert, coffee and roast pork with all the trimmings.”* (M, 60)  *“When the children come home at Easter, I have to cook something else [...], then we have Wiener schnitzel.“* (F, 65)  *“And also at the wedding, for example, the only thing [vegetarian] was the baked mushrooms.“* (M, 60) |

| **ENVIRONMENTAL LEVEL** |
| --- |
| **Theme: Eating out** |
| *“Not eating meat isn't really a problem at all now, it's only when you're, like, invited to a restaurant or something […] that there's nothing really vegetarian on the menu.”* (M, 60)  *“Well, I actually look which vegetarian food is on the menu, and if it appeals to me, I order that.”* (F, 69)  *“But I don't think that's a problem, because on holidays, for example, I had fish almost every day.”* (F, 68)  *“On holiday, it's only possible in very expensive hotels that you really have this good selection. Normally, not so much, this is something I noticed, so I find that difficult.”* (M, 66)  *“Choosing from the buffet, starting with the salad and vegetable side and then moving on to the meat. So more or less against the flow, so there wasn't so much room on the plate [for meat].“* (M, 66)  *“If I’m going out for a meal or meeting someone, then I eat what I don’t eat at home.”* (F, 65)  *“You’re happy that you don’t have to cook by yourself [note: at invitations] and eat what is on the table.”* (F, 61)  *“I noticed that it [note: following the diet] works less well […] e.g., at an invitation to a barbecue.”* (M, 66) |
| **Theme: Food access and purchasing decisions** |
| *“For me, changing my diet definitely starts with shopping. If I don't have the stuff at home, then I can do without it quite easily.”* (M, 66)  *“If we have the right ingredients at home, then we can always cook something from them, and if this is what we bring home, then it's what we have and what we cook.”* (F, 62)  *“We probably have, I don't know, regional shops somewhere, but not in Vienna where I work, so I just go to Billa [note: supermarket].”* (F, 58)  *“The main focus is always locally sourced, the second focus is organic.”* (F, 52)  *“And basically, if you buy higher-quality food, then it's more expensive.“* (M, 66)  *“Shopping at the farmer's is always more expensive than at Spar or Hofer [note: supermarkets].”* (M, 66)  *“I mean, with the pulses, I’ve never actually cooked them […] this year I’ve grown a lot in the garden, so I will cook them for sure.”* (F, 68)  *“I cook differently in summer than in winter, so in summer I cook things from the raised bed and in winter I cook these lentils and beans and all that.”* (F, 62)  *“It’s only now that I realize what a treasure we can grow ourselves. In the last three or four weeks, I think we've had lettuce every day […] cut off directly with a knife.”* (M, 58)  *“I also did very well during the changeover – at least after I had used up everything that was still there.“* (F, 61)  *“Just get rid of what you have at home and then go shopping. Yes, and choose different things.“* (F, 61) |
